# PDE4-Selective Inhibition in Chronic Obstructive Pulmonary Disease and Pulmonary Fibrosis: Different Agents or Different Targets?

**DOI:** 10.3390/life15101600

**Published:** 2025-10-14

**Authors:** Graeme B. Bolger

**Affiliations:** BZI Pharma LLC, 100 Cummings Center, Suite 241-D, Beverly, MA 01915, USA; graemebolger@bzipharma.com or gbbolger@uab.edu

**Keywords:** COPD, IPF, PPF, roflumilast, apremilast, nerandomilast, cAMP, phosphodiesterase, treprostinil

## Abstract

Highly selective inhibitors of the members of the cAMP-selective cyclic nucleotide phosphodiesterases, or PDE4 family, have shown clinically meaningful activity in two different classes of lung disease: roflumilast in obstructive lung disease, specifically chronic obstructive pulmonary disease (COPD), and nerandomilast in restrictive lung diseases characterized by inflammation/fibrosis of the alveolar interstitium, including idiopathic pulmonary fibrosis (IPF) and progressive pulmonary fibrosis (PPF). The beneficial therapeutic benefit of these agents in both of these disorders suggests that they share a common mechanism that underlies their effects on different pulmonary cells and tissues. This review outlines the biochemical, pharmacologic and cellular effects of PDE4-selective inhibitors, emphasizing their role in signal transduction pathways common to many pulmonary cell types. It then compares and contrasts the myriad cellular effects of these agents and their effects in pre-clinical animal models of these disorders. The emerging data are compatible with PDE4-selective inhibitors having targets of action in a large number of pulmonary cell types, only a subset of which is dysregulated in either COPD or IPF. This suggests that differences between the benefits observed with these individual agents in their various clinical indications reflect differences in disease pathogenesis, rather than proven differences in the enzyme-inhibitory effects of the various PDE4 inhibitors that have been studied to date.

## 1. Introduction

Highly selective inhibitors of the cAMP-specific phosphodiesterases, or PDE4 enzymes, have been studied intensively in numerous, rigorous clinical trials and are currently available for clinical use in a wide variety of human diseases, where they have potent immunomodulatory and anti-inflammatory effects (Table 1; refs. [1,2]). PDE4-selective inhibitors have been conspicuously tested in a range of pulmonary disorders, including chronic obstructive pulmonary disease (COPD) and pulmonary fibrosis, where much of their clinical efficacy reflects their anti-inflammatory actions [3,4,5,6,7]. PDE4-selective inhibitors also have numerous other effects in the lung, including the modulation of ion channel activity ([8], see ref. [9] for a review) and potential effects on the modulation of airway smooth muscle tone.

The expansion of the PDE4-selective toolbox in human diseases, especially in the lung, currently reveals an interesting paradox. Many of the currently available PDE4-selective inhibitors have overlapping, indeed sometimes indistinguishable, enzyme-inhibitory profiles, as described in more detail below. However, review of the current clinical indications for these drugs shows that each is currently indicated for a narrow range of disorders that typically has minimal, if any, overlap with the indications for the other available drugs in this class (Table 1).

This review will discuss to what extent these therapeutic differences reflect differences in the mechanistic effects of these drugs, with special emphasis on pulmonary cells and tissues. It will start with a review of the clinical and biologic aspects of COPD and pulmonary fibrosis, including recent attempts to define sub-classes of these disorders that would guide therapeutic management. It will then discuss pre-clinical models of each of the disorders in an attempt to characterize their pathophysiology. With this foundation in place, it will review the biochemical and cellular mechanisms of action of PDE4 inhibitors in these disorders. It will then turn directly to clinical issues, including completed and ongoing clinical trials of these agents. Finally, it will offer a perspective on biomarker development in relation to both clinical practice and the next generation of clinical trials.

## 2. COPD and Pulmonary Fibrosis: Similarities and Differences in Clinical Presentations and Therapy

Although PDE4 inhibition has therapeutic value in both COPD and fibrotic lung disease, these two classes of lung disorders differ profoundly in their pathophysiology and clinical manifestations.

### 2.1. COPD—Definition and Sub-Classes

According to the 2025 report from the Global Initiative for Chronic Obstructive Lung Disease (“GOLD”), COPD is defined by chronic respiratory symptoms caused by airway abnormalities that produce persistent and frequently progressive airflow obstruction [21]. The diagnosis is confirmed by the demonstration of non-fully reversible airflow obstruction on spirometry [21]. Patients typically complain of dyspnea and/or cough. Examination of lung tissue from patients with COPD shows small-airway narrowing (bronchiolar obstruction), destruction of alveolae and their replacement with large air-filled cavities (emphysema), increased mucus production, inflammation and fibrosis [22]. These pathologic findings are consistent with inflammation in COPD leading to tissue destruction; in contrast, in pulmonary fibrosis, inflammation leads to abnormal cellular proliferation.

The heterogeneity of COPD, even for patients whose disease clearly conforms to the GOLD criteria, has stimulated attempts to divide the disease into sub-types. The 2025 GOLD report proposes a taxonomy based on differences in etiology (“etiotypes”), including, for example, genetically determined causes, or environmental causes (smoking, pollution), or prior infections or asthma [21]. Alternatively, patients can be subdivided into groups on the basis of clinical findings. Such a clinically based classification could identify patients with a predominantly bronchitic presentation (i.e., the regular production of sputum for several months each year [23]), or, alternatively, those presenting predominantly with emphysema (airway obstruction and structural lung lesions on imaging). An increased level of circulating eosinophils has been used to define a subtype of COPD that has a strong allergic/inflammatory component and whose pathophysiology that overlaps that of asthma; such patients do not necessarily have the reversible airway obstruction characteristic of asthma, but typically benefit from some of the anti-inflammatory strategies that help patients with asthma, such as inhaled corticosteroids [24,25,26,27,28,29].

Extensive clinical study has shown the value of roflumilast and several other PDE4-selective inhibitors, such as cilomilast, in COPD, even though they do not have significant acute bronchodilator activity (reviewed in Refs. [30,31]). Roflumilast has shown to reduce the frequency of COPD exacerbations, especially in patients with severe COPD who have chronic bronchitis and/or are repeatedly hospitalized for COPD [5,10,11,12,32]. Clinical trials have shown roflumilast to be of value in patients who cannot tolerate inhaled corticosteroids or who are not well-controlled on inhaled LABA/LAMA/steroid combinations [12,31]. Nausea, diarrhea, and changes in mood and behavior, all typical class side effects of PDE4 inhibitors, lead to its discontinuation in some patients [5,30].

The development of ensifentrine, the first PDE inhibitor that can be administered by nebulization, is an important advance in the therapy of COPD, especially for patients presenting with COPD exacerbations [17,33,34,35,36,37]. Ensifentrine has activity against both PDE3 and PDE4 [38]; see ref. [9] for a review. A slightly older inhaled PDE4 inhibitor, tanimilast, has also shown activity in several clinical trials (see ref. [9] for a review) but has not been approved for clinical use. Unlike roflumilast or nerandomilast, ensifentrine has significant acute bronchodilator activity, which contributes substantially to its therapeutic action.

Patients with COPD and eosinophilia have been shown to benefit from monoclonal antibodies that target elements of the immune system, such as dupilumab [39,40,41], or, very recently, mepolizumab [42,43]; see refs. [44,45] for reviews. Dupilumab blocks a shared component of the receptors for interleukin-4 and interleukin-13 (IL-4 and IL-13; refs [46,47]), while mepolizumab targets interleukin-5 (IL-5; refs [43,48]). IL-4, IL-5 and IL-13 are key drivers of Type 2 inflammation, key to the pathogenesis of many disorders associated with eosinophilia ([49,50]; see ref. [51] for a review).

As the number of potential therapeutic options expands in COPD, there has been a pressing need to develop better classification systems that would inform clinical practice and the design of the next generation of clinical trials (see ref. [9] for a review). For example, patients with a bronchitic phenotype would seem to benefit from inhaled β2-adrenergic agonists and muscarinic antagonists. They will also generally benefit from roflumilast and/or ensifentrine. Patients with eosinophilia will benefit from the addition of dupilumab or mepolizumab to their ongoing inhaled corticosteroids. There remains a large number of COPD patients, typically those predominantly with emphysema, who lack therapeutic options beyond inhaled muscarinic antagonists. Further research on the pathobiology of COPD may yield additional prognostic/classification criteria, especially additional biomarkers, as discussed in greater detail below.

### 2.2. Pulmonary Fibrosis—Definition and Sub-Classes

Pulmonary fibrosis can be defined as the development of pulmonary lesions characterized by fibroblast proliferation in the alveolar interstitium that is associated with progressive pulmonary dysfunction [52]. Idiopathic pulmonary fibrosis (IPF), i.e., fibrosis that is not associated with an underlying medical disorder, is a subset of interstitial lung disease (ILD), a broader class of pulmonary disorders that is characterized by inflammation and/or fibrosis of the lung parenchyma [53]. Progressive pulmonary fibrosis (PPF) describes a fibrosing ILD that arises in pre-existing pulmonary disease, such as auto-immune ILD, hypersensitivity pneumonitis, or other forms of ILD [18]. Typical auto-immune disorders leading to PPF are scleroderma, rheumatoid arthritis with systemic manifestations, and other rheumatologic conditions [54]. PPF can be produced by exposure to numerous drugs, including amiodarone and several anti-neoplastic agents, especially bleomycin [55]. Finally, there is a well-validated association between germline variants (single nucleotide polymorphisms, or SNPs) in the telomerase genes *TERT* and *RTEL1*, as well as weaker associations with some other loci, and the development of IPF and other fibrosing diseases [56,57,58,59]. Closely related to IPF and PPF are pulmonary diseases associated with exposure to particulates, such as asbestosis [60] and silicosis [61,62]. Although there is yet no formal classification of pulmonary fibrosis that includes all of the above conditions, current clinical practice includes many of the above factors in decision-making, and, more recently, in the design of clinical trials [18,19,52].

Two agents are currently available for the treatment of pulmonary fibrosis; on the basis of recently completed phase 3 clinical trials, another two are likely to be approved very shortly (Table 2).

Pirfenidone, the first agent to be developed specifically for fibrotic lung disease, has been shown on the basis of multiple phase 3 trials to be safe and effective in both IPF and PPF [68,69,70,71]. The biochemical target of pirfenidone has been the subject of considerable research; it is clearly not a PDE4-selective inhibitor (see references in the pre-clinical sections below). Nintedanib, which targets several protein-tyrosine kinases, most convincingly the receptors for vascular endothelial growth factor (VEGF), platelet-derived growth factor (PDGF), and fibroblast growth factor (FGF), has also been shown on the basis of multiple phase 3 trials to be safe and effective in both IPF and PPF [63,64,65,66]. Combination therapy with both of these agents appears to have additive benefit [72,73,74].

The PDE4B-selective inhibitor nerandomilast has been shown on the basis of two phase 3 trials to be safe and effective in both IPF and PPF [19,67]. Head-to-head comparisons of nerandomilast with older agents in fibrotic lung disease have yet to be reported, but the magnitude of benefit seen with nerandomilast in these two phase 3 trials appears to be as good as, or possibly better than, that of the older drugs [75,76,77].

Several recent phase 3 clinical trials, some of which have yet to be published in full, have demonstrated activity of the prostaglandin analog treprostinil in patients with IPF [78,79,80,81,82]. These trials have tested treprostinil in at least two different formulations as a dry powder (i.e., as a metered-dose inhaler) and, more recently, in an oral form. This drug has been available for some time for the treatment of pulmonary arterial hypertension (PAH); the newer study studies its effects in patients with both PAH and IPF. Treprostinil acts primarily as a vasodilator; its benefits in IPF may be due to its vasodilatory properties. However, given the key role of prostaglandins in the pathogenesis of IPF (see below), it may also have beneficial effects on the underlying disease process.

Given the considerable differences between COPD and the fibrotic lung diseases, it is perhaps surprising that both these classes of disorders can benefit from treatment with PDE4-selective inhibitors. To determine what aspects of PDE4 inhibition might be responsible for this common effect, it is useful to review the enzymology, pharmacology and cellular regulation of the PDE4s and recent advances in PDE4 inhibitor pharmacology.

## 3. Molecular Pharmacology of the PDE4 Family: Genes, mRNAs, and Proteins

### 3.1. cAMP Signaling

**Figure 1:**
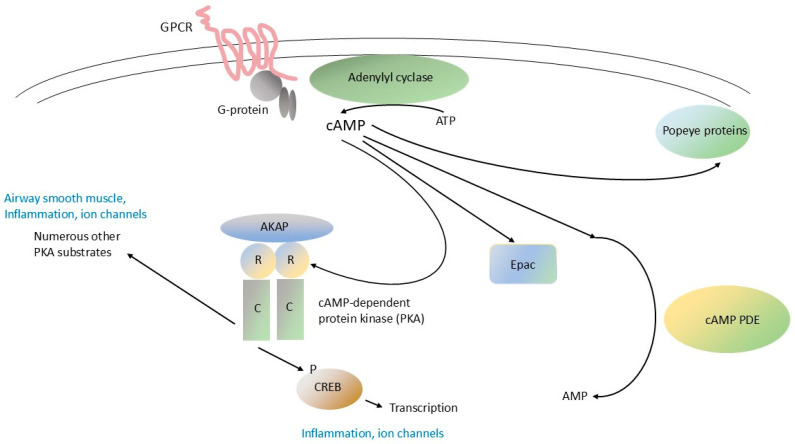
Signal transduction pathways mediated by cAMP in respiratory cells: role of PDE4 cyclic nucleotide phosphodiesterases. G-protein-coupled receptors (GPCRs) are a large family of 7-helix transmembrane proteins that are the physiological receptors for numerous circulating hormones and neurotransmitters and also ligands for myriad small-molecule drugs. GPCRs are controlled by small molecules that bind to their extracellular or transmembrane regions. One GPCR that plays an important role in pulmonary physiology is the β_2_-adrenergic receptor (β2-AR), the target for β-adrenergic agonists. Another important set of GPCRs in the lung are those for prostaglandins, such as PGE_2_. GPCRs interact with trimeric G-proteins by recruiting them to specific regions on their intracellular loops. G-proteins have three subunits (α, β, and γ) and the members of the α subunit family can be divided into stimulatory (Gsα) or inhibitory (Giα) isoforms. Agonist binding to GPCRs can cause them to interact with, and activate, Gsα. Activated Gsα stimulates various membrane-associated adenylyl cyclase (AC) isoforms, which catalyze the synthesis of cAMP. cAMP is a soluble “second messenger” that can diffuse widely in cells but whose concentration is often tightly regulated within specific sub-cellular compartments. cAMP PDEs, notably members of the cAMP-specific PDE, or PDE4 family, selectively hydrolyze cAMP and thereby regulate its levels in cells. The downstream effectors of cAMP include cAMP-dependent protein kinase (PKA), exchange protein activated by cAMP (EPAC) and Popeye proteins. PKA is anchored to specific subcellular locations through its binding to A-kinase anchoring proteins (AKAPs). Binding of cAMP to the PKA regulatory (R) subunits activates the PKA catalytic (C) subunits, which phosphorylate (P) serines and threonines located in clearly defined regions of numerous PKA substrate proteins. A major PKA substrate is the cystic fibrosis transmembrane conductance regulator (CFTR), which acts as a Cl^−^ ion channel. Another important PKA substrate is the loop-helix-loop transcription factor, cyclic nucleotide response-element binding protein (CREB). Phosphorylation of CREB by PKA and other kinases causes it to dimerize and translocate to the nucleus, where it binds to specific cyclic AMP response elements (CREs) in active chromatin, thereby regulating the transcription of numerous genes. The thin curved lines near the top of the figure represent the apical plasma membrane.

Signal transduction pathways modulated by the prototypical small molecule cyclic nucleotide “second messenger” 3′, 5′ cyclic adenosine monophosphate (cAMP) are ubiquitous in cells and tissues. cAMP signaling plays a critical role in the function of diverse organs, including the lung, the CNS, inflammation and immunity, and the endocrine system. cAMP is synthesized by membrane-associated adenylyl cyclase in response to extracellular stimuli, and then diffuses throughout the cell, where it interacts with specific downstream effector proteins (Figure 1, ref. [3]). G-protein-coupled receptors (GPCRs), which regulate membrane-associated adenylyl cyclase through trimeric GTP-binding proteins (G-proteins) are among the most important regulators of adenylyl cyclase and thereby cAMP signaling generally. Also essential to the regulation of cAMP signaling are cyclic nucleotide phosphodiesterases (PDEs), which are enzymes that hydrolyze (degrade) cAMP (and/or 3′, 5′ cyclic guanosine monophosphate, or cGMP) and thereby modulate its levels in cells [3,4,7,83]. Downstream effectors of cAMP action in cells include cAMP-specific protein kinase (protein kinase A, PKA), cyclic nucleotide-gated ion channels, exchange proteins activated by cAMP (EPACs), and Popeye proteins [84,85,86,87,88]. These various pathway components also mediate “cross-talk” between cAMP signaling and other cellular pathways, particularly the MAPK pathway. Both synthesis and breakdown of cAMP can be highly localized in cells, producing “compartments”, “pools” or “gradients” where its concentration is tightly regulated in space and time [89,90,91]. The overall process is highly dynamic, with short- and long-term feedback loops that adjust the “gain” of various components of the pathways and increase their versatility and range of response.

### 3.2. The PDE Superfamily

The ability of the cAMP-specific phosphodiesterases, or PDE4 enzymes, to hydrolyze cAMP is key to their role in regulating cAMP signaling pathways in cells. The PDE4s are members of the larger cyclic nucleotide phosphodiesterase superfamily, whose members consist of a total of 11 families, encoded by a total of 21 genes [1,3,83]. The various members of the PDE superfamily are defined on the basis of their substrate specificity (cAMP, cGMP, or dual-specificity for both cAMP and cGMP) and their ability to be inhibited by pharmacologic inhibitors specific to each family. PDE3, PDE4, PDE7 and PDE8 are cAMP-selective; while PDE1, PDE2, PDE10 and PDE11 are dual-specific [3,4,7,83]. The primary amino acid sequence and fold of the catalytic regions of each family determine, at least in part, the substrate specificity of each family member and its ability to be selectively inhibited by family-specific inhibitors [83,92,93]. Consistent with this observation, PDE-selective inhibitors act at the catalytic sites of the PDE enzymes and therefore act, at least in part, as competitive inhibitors of cAMP and/or cGMP hydrolysis. To date, PDE4 and possibly PDE3 have been shown to be pharmacologically relevant targets in COPD (see references above and refs. [1,9] for reviews) and fibrotic lung disease (see references above). PDE5 inhibitors modulate nitric oxide—cGMP signaling and have been shown to be safe and effective in the treatment of PAH. The pharmacologic potential of other PDE families in pulmonary disorders has yet to be determined.

### 3.3. PDE4 Multiplicity: Genes, mRNAs and Proteins

**Figure 2:**
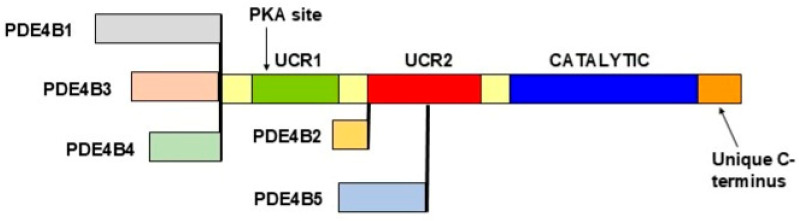
Schematic of human PDE4B isoforms. The long isoforms PDE4B1, PDE4B3, and PDE4B4 contain UCR1 and UCR2, the catalytic domain, plus isoform-specific unique regions at their amino-termini. The short isoform PDE4B2 contains the catalytic domain and UCR2, while the super-short PDE4B5 isoform contains the catalytic region and a portion of UCR2. UCR1 and UCR2 mediate dimerization of the long PDE4B isoforms. Also shown are the carboxyl-terminal region, common to all PDE4B isoforms, and the PKA site located within UCR1. The various PDE4B isoforms are all generated by the use of alternative mRNA splicing and the use of alternative promoters located within the *PDE4B* gene. The nomenclature of PDE4 isoforms is therefore based on gene name (*PDE4A* vs. *PDE4B* vs. *PDE4C* vs. *PDE4D*), with each individual isoform identified by a number (e.g., PDE4B1 vs. PDE4B2).

Key to understanding the effects of PDE4 inhibitors in tissues and organs is the incredible diversity of PDE4 isoforms. There are over 20 PDE4 isoforms, which are encoded by 4 genes in mammals (*PDE4A*, *PDE4B*, *PDE4C* and *PDE4D* in humans), with additional diversity being produced by alternative mRNA splicing and the use of alternative promoters for each isoform [94,95]. Specifically, the human *PDE4B* gene encodes 5 different isoforms (Figure 2; see ref. [96] for a review) and the human *PDE4D* gene encodes 9 different isoforms (see ref. [97] for a review). The mRNA, and corresponding protein, for each isoform has a distinct pattern of expression in tissues, suggesting that each has a distinct tissue or organismal function; these cellular and regional differences in expression has been explored most carefully in the central nervous system (see refs. [96,97] for a review), but is highly likely to be the case in other organs, including the lung.

PDE4 proteins can consist of up to three domains, each of which has a distinct primary sequence and fold. The catalytic region contains all elements necessary for cAMP hydrolysis, while the UCR1 and UCR2 regions are essential for dimerization and serve to regulate the activity of the catalytic domain (Figure 2, refs. [94,98]). “Long” PDE4 isoforms contain UCR1, UCR2 and the catalytic domain, while “short” isoforms contain UCR2 and the catalytic domain, and “super-short” isoforms contain a portion of UCR2 and the catalytic domain. The catalytic sites of the various PDE4 isoforms are extremely similar (approximately 90% primary amino acid sequence homology, refs. [94,95]); until relatively recently, this close homology complicated the development of inhibitors selective for the isoform(s) encoded by any specific PDE4 gene.

### 3.4. Regulation of the PDE4 Proteins

The various PDE4 isoforms are regulated by a complex and interacting network of phosphorylation events and interactions with other signaling proteins (see refs. [1,4,95]). Numerous proteins have been shown to interact with specific PDE4 isoforms: for example, the DISC1 protein interacts specifically with long PDE4B isoforms, most notably PDE4B1 [99], while the RACK1 protein and β-arrestin2 both interact specifically with PDE4D5 [100,101]. The subcellular localization of numerous PDE4 isoforms is determined by their interaction with scaffold and anchoring proteins, including A-kinase anchoring proteins, or AKAPs [102]. These and other protein–protein interactions can determine the subcellular localization of various PDE4 isoforms, their ability to undergo phosphorylation (see next paragraph), and their interactions with other signal transduction components [1,4,7].

Among the most important mechanisms for the regulation of PDE4 isoforms is their ability to be phosphorylated by several physiologically important kinases. Long PDE4 isoforms are phosphorylated by PKA at a specific serine located at the N-terminal end of UCR1 (Figure 2); PKA phosphorylation serves to inhibit the activity (velocity) of the enzyme [103,104]. PKA phosphorylation also is essential to the dimerization of long PDE4 isoforms, which may be essential for enzyme inhibition [98]. Through dimerization or potentially other mechanisms, PKA phosphorylation changes the conformation of the catalytic region, as manifested by changes in the susceptibility of long PDE4 isoforms to PDE4-selective inhibitors [95,103]. Germline mutations in the human *PDE4D* gene that localize to UCR1 and UCR2 and which affect dimerization and PKA regulation of long PDE4D isoforms cause acrodysostosis, a debilitating disorder that affects bone formation and the brain (ref. [105]; see ref. [83] for a review). PDE4 isoforms are also phosphorylated by ERK1/2 and undergo numerous other forms of secondary modification; determining the physiological significance of many of these events remains an active area of investigation [106,107].

### 3.5. Structure and Regulation of the PDE4 Proteins: Relevance to Roflumilast and Nerandomilast

Given our basic knowledge of the structure and regulation of the PDE4 enzymes, what insights can this knowledge provide on the actions of roflumilast and nerandomilast? The available pre-clinical data does suggest that there is a difference in the selectivity of these two drugs. Roflumilast and its active metabolite, roflumilast N-oxide, are highly specific for PDE4 (as opposed to other PDE families), but inhibit all PDE4 isoforms relatively equally (i.e., IC_50_ values that vary from 0.2 to 7.8 nM, depending on the PDE4 isoform, ref. [8]). In contrast, the values provided for nerandomilast show it to be relatively specific for PDE4B (IC_50_ values of 10 nM for PDE4B, 91 nM for PDE4D, 248 nM for PDE4A, and 8700 nM for PDE4C, ref. [108]. However, it should be noted that the enzyme preparations used in the reported nerandomilast assays consisted of PDE4B and PDE4D active site fragments (i.e., containing just the core catalytic domain (Figure 2)), while those for PDE4A appeared to include UCR1, UCR2 and the catalytic region, and those for PDE4C apparently included full-length protein (PDE4C2; ref. [108]). Given that UCR1 and UCR2 have important effects on the activity of the PDE4s, including their ability to dimerize and be phosphorylated by PKA and other kinases, it is quite possible that different values for specificity would have been obtained if full-length isoforms had been compared [109]. More generally, in the future, it will be of considerable value if comparative IC_50_ values are obtained on correspondingly similar enzymes (e.g., by comparing the long isoforms encoded by each of the 4 different PDE4 genes, OR by comparing just the isolated catalytic domain for isoforms encoded by the 4 different PDE4 genes). Finally, given the important effects of phosphorylation on the activity of PDE4s, and on their ability to be inhibited by PDE4-selective inhibitors, it would be essential to examine/compare phosphorylated and non-phosphorylated long enzyme preparations.

Given these insights into how roflumilast and nerandomilast act on the enzymatic aspects of the PDE4 enzymes, with special emphasis on the PDE4B enzymes, it is of particular interest how they might influence the cellular and tissue effects of these drugs. This will be explored in the next two sections.

## 4. Defining the Cellular Targets of PDE4 Inhibitors: Insights from Single-Cell Sequencing

Advances in single-cell RNA sequencing have greatly enlarged knowledge of the various cell types present in the lung, and in turn have increased the precision in determining the site of action of PDE4-selective inhibitors. Single-cell sequencing of human and murine tracheal epithelium has demonstrated the presence of many new cell types (58 in all), many of which were previously not previously identified [110]. Among the new cell types identified by these studies are pulmonary ionocytes [111,112], which are highly enriched in the cystic fibrosis transmembrane regulator (CFTR) protein. CFTR is a Cl^−^ ion channel that is regulated, at least in part, by cAMP-signaling pathways, which modulate the phosphorylation of the CFTR regulatory domain by PKA. Several laboratories, including our own, have shown that PKA-mediated phosphorylation of CFTR is in turn regulated by PDE4 (see ref. [9] for a review). More generally, the expanded knowledge of the cell types, and the gene-expression pattern in each of these cell types, in the lung has the potential to greatly increase our ability to determine which cell types in the lung are the targets of pharmacological intervention. We apply these new concepts to PDE4 action in the lung in the next sections.

## 5. Pre-Clinical Models of COPD and Fibrotic Lung Disease: Clues to the Mechanisms of PDE4 Action in Lung Diseases

### 5.1. Pre-Clinical Models of COPD Demonstrate Multiple Beneficial Effects of PDE4 Inhibition in COPD

Extensive clinical study has shown the value of roflumilast and several other PDE4-selective inhibitors, such as cilomilast, in COPD. PDE4 inhibitors do not have significant acute bronchodilator activity in COPD ([113], see ref. [8] for a review). However, they have myriad effects on pulmonary cells and tissues that collectively account for their therapeutic benefit.

### 5.2. Role of PDE4 in Pulmonary Inflammation

Decades of pre-clinical studies have demonstrated that PDE4-selective inhibitors have activity in various immune/inflammatory pathways in lung tissue. The extensive data for first-generation PDE4-selective inhibitors has been summarized in older reviews [95]. Many of the older studies focused on models pertinent to allergy/asthma that are of uncertain relevance to COPD. Roflumilast and its active metabolite, roflumilast N-oxide, affects the functions of many cell types, including CD4+ and CD8+ T-cells, monocytes/macrophages and neutrophils [8]. In particular, the action of roflumilast against neutrophils probably explains much of its activity in COPD, as neutrophilic airway inflammation is a hallmark of COPD, as described below. In contrast, the monoclonal antibodies dupilumab and mepolizumab appear to act on inflammatory processes mediated, at least in part, by eosinophils [43,46,47,48,49,50,51]. The importance of neutrophil inflammation in COPD also appears to explain much of the pathogenesis of the disease, specifically the role of neutrophil-derived proteases, especially matrix metallopeptidases, and reactive oxygen species, in the destruction of lung tissue typically of COPD.

### 5.3. Other Actions of Roflumilast in the Lung

Roflumilast has actions on numerous non-immune/inflammatory cells in the lung, including airway and vascular smooth muscle, endothelial cells, and respiratory epithelium. Its actions on vascular smooth muscle cells probably account for its ability to prevent or ameliorate pulmonary hypertension in several rodent models [8] and potentially in humans. Roflumilast also has potent effects on fibroblasts, as discussed in detail below. Roflumilast is a potent stimulator of the CFTR Cl^−^ ion channel, which is expressed in pulmonary epithelial cells and ionocytes and which is essential for mucociliary clearance and the maintenance of airway surface liquid (see ref. [9] for a review). Collectively, these immune and non-immune effects of roflumilast are likely to account for its beneficial action in mouse models of COPD, and in clinical use.

### 5.4. Actions of Roflumilast in Animal Models of COPD

Extensive study of the effects of roflumilast in rodent models of COPD have provided insights into its cellular, tissue and whole-organ benefits in human COPD. The rodent models used in these experiments have a number of limitations and certainly do not replicate all aspects of human COPD. The most-commonly used model is the treatment of rodents, typically mice, with cigarette smoke for a few hours or days; this model clearly differs substantially from COPD in humans, which typically progresses over many years. A variant animal model, incorporating smoke exposure for several months, would appear to be more appropriate, but even the mice in this model develop only the early stages of COPD (GOLD stages I and II; see ref. [8] for a review). However, these models show clearly that smoke exposure produces primarily a neutrophilic inflammatory response [8]; this may account for the beneficial effects of roflumilast and other PDE4-selective inhibitors in these models. In contrast, corticosteroids, which have beneficial effects in many forms of airway inflammation, especially in asthma [51], are ineffective in these models. Although additional refinement of these models would be useful, they provide a useful platform for the testing of compounds that can be moved into clinical trials. Further study of these models also has the potential to accelerate biomarker development, which is a key unmet clinical need in COPD.

### 5.5. Pre-Clinical Models of Fibrotic Lung Disease and the Role of PDE4 Inhibition

Key to understanding fibrotic lung disease, including IPF and PPF, is the proliferation of fibroblasts, which form the cellular backbone of the lesions seen in these disorders. Fibroblasts also synthesize and secrete extracellular molecules, such as collagen and other components of the extra-cellular matrix, which are essential components of these lesions. Although fibroblast proliferation in disease can be the end-result of various stimuli, it is generally agreed that inflammation is a key contributing factor and that fibroblast proliferation mediated by the production of various growth factors and cytokines by inflammatory cells, especially neutrophils, is a hallmark of PPF and IPF. PDE4 inhibitors can intervene at several steps in this process.

### 5.6. PDE4s and Fibroblast Proliferation in the Human Lung

Roflumilast, nerandomilast, and other PDE4-selective inhibitors have anti-fibrotic activity in several cell-based models of inflammation-mediated fibrosis. A commonly used model assesses the deformation of collagen gels in in-vitro culture, where fibroblast proliferation produces complex three-dimensional structures. Roflumilast can slow fibroblast growth and attenuate the development of these structures in these cultures. In these models, roflumilast acts synergistically with prostaglandin E_2_ (PGE_2_), which binds to its cell-surface receptor, a GPCR, on fibroblasts, activates adenylyl cyclase, and increases intracellular cAMP (Refs. [8,54,114,115,116,117,118,119,120], Figure 1). PGE_2_ can be derived from activated neutrophils or macrophages, or be produced by proliferating fibroblasts and act in an autocrine fashion. Fibroblast proliferation mediated by the production of VDGF, PDGF and FGF by inflammatory cells is also an important contributor to this process. Another key regulator of fibroblast proliferation is transforming growth factor beta 1 (TGF-β1), which is secreted by macrophages and other inflammatory cells and which acts through SMAD pathway signaling to stimulate fibroblast proliferation and production of extra-cellular matrix proteins [108,121,122,123,124]. Tumor necrosis factor alpha (TNF-α), which is produced primarily by neutrophils and activated macrophages [108,120,125,126,127] and which has numerous effects on immune effector cells, also stimulates fibroblast growth and the production of extracellular matrix. It is likely, therefore, that roflumilast, nerandomilast, and other PDE4-selective inhibitors have a dual mechanism of action in these models: (1) they have direct effects on fibroblasts and (2) they down-regulate immune/inflammatory cells that produce factors that stimulate fibroblast growth.

### 5.7. Actions of Roflumilast and Nerandomilast in Animal Models of Fibrotic Lung Disease

Several well-validated rodent models have provided key insights into the pathogenesis of human fibrotic lung disease and also serve as test platforms for assessing PDE4 inhibitors (and other treatments) in these disorders. The best-described model is bleomycin-induced lung fibrosis in mice. Bleomycin is an anti-neoplastic agent with potent activity in several human cancers; it is a potent generator of free radicals, which accounts for both its antitumor activity and its toxicities. Intra-tracheal treatment of mice with bleomycin produces an intense inflammatory response that is rich in macrophages, neutrophils and lymphocytes and then, over several weeks, progresses to severe fibrosis. TNF-α, along with other cytokines, is felt to be a major contributor to bleomycin-induced lung inflammation and fibrosis. Bleomycin-induced lung inflammation is attenuated by corticosteroids, which also prevent or ameliorate the resulting fibrosis [128]. In contrast, as described above, tobacco smoke typically produces lung inflammation that does not progress to fibrosis and which does not respond to glucocorticoids [8].

All three oral drugs (pirfenidone, nintedanib, and nerandomilast) currently available for the treatment of IPF and PPF have significant activity in bleomycin-induced mouse lung injury models [108,129,130,131,132,133,134,135], and pirfenidone and nintedanib have been used successfully off-label for the treatment of bleomycin lung toxicity in humans [55]; because nerandomilast has only recently been approved, clinical use in human bleomycin toxicity has yet to be reported. Roflumilast also has significant activity in the same mouse model [128,136,137] and has also been used in humans therapeutically for this purpose [55]; head-to-head comparisons between roflumilast and nerandomilast (or other agents) in this setting has yet to be reported.

Apart from bleomycin, exposure to several other agents, including the minerals silica and asbestos, can also produce fibrotic lung disease in mouse models (and also in humans); there is less data on the value of pharmacological interventions in these models [60,61,108].

### 5.8. PDE4 Expression and Function in Key Pulmonary Cell Types: Relationship to COPD and Pulmonary Fibrosis

Although there are extensive data from pre-clinical models for the efficacy of both roflumilast and nerandomilast in both COPD and pulmonary fibrosis, important questions remain. What specific PDE4 isoforms (e.g., those encoded by *PDE4A* vs. *PDE4B* vs. *PDE4D*, and the various isoforms encoded by each of these genes) are responsible for the effects of both of these drugs in these models? There is surprisingly little reported data on which PDE4 isoforms are expressed and functionally active in each of the pre-clinical models; such knowledge would allow the generation of hypotheses about how they modulate specific PDE4 signaling pathways. Further characterization of these models, especially using the newer technology of single-cell mRNA sequencing, would be very valuable. The current state of knowledge is summarized in Table 3.

In respiratory epithelium and pulmonary ionocytes, PDE4 isoforms are essential modulators of CFTR Cl^−^ channel activity and thereby play an important role in the maintenance of airway surface liquid. PDE4 isoforms are also important modulators of mucus secretion and ciliary function, both of which, along with airway surface liquid, contribute to pulmonary epithelial mucociliary clearance. The release of reactive oxygen species (ROS) and various inflammatory cytokines, such as TNF-α, IL-1 and IL-8, by pulmonary neutrophils is also regulated, at least in part, by various PDE4 isoforms. The production of TNF and other cytokines, and the release of inflammatory mediators and growth factors, by macrophages, neutrophils and fibroblasts in lung tissue also reflects regulation of these functions, at least in part, by PDE4 isoforms. Finally, PDE4 isoforms are present and have important function roles in lymphocytes that have migrated into lung tissue.

Given our current state of knowledge, it is clear that further pre-clinical research is necessary to determine the exact role(s) of individual PDE4 isoforms (e.g., those encoded by *PDE4A* vs. *PDE4B* vs. *PDE4D*, and the various isoforms encoded by each of these genes) in critical aspects of the pathogenesis of COPD, such as small airway inflammation, mucus hypersecretion, the activation and tissue effects of neutrophils and macrophages, and the production of various cytokines. Similarly, much more pre-clinical research is necessary for determining the roles of individual PDE4 isoforms in critical aspects of the pathophysiology of pulmonary fibrosis, such as the migration, proliferation and differentiation of fibroblasts, the activation of the TGF-β pathway in these cells, and the role of fibroblasts in production and remodeling of the extracellular matrix. Data obtained from these studies will provide better pre-clinical rationale for the use of roflumilast in COPD and nerandomilast in pulmonary fibrosis, respectively.

## 6. Insights from Clinical Investigation, Clinical Trials, and Translational Research

### 6.1. Lessons from Clinical Trials of Roflumilast in COPD; Role of Inflammation

The clinical data on roflumilast clearly confirms the pre-clinical data that a major target of the drug is PDE4 isoforms in pulmonary inflammatory cells, as opposed to PDE4s in vascular or airway smooth muscle. Early-stage clinical trial data with roflumilast showed that it did not have significant acute bronchodilator activity in COPD ([113], see ref. [8] for a review). Consequently, phase 3 clinical trials of roflumilast have focused on patients with COPD and severe airflow limitation, symptoms of bronchitis, and frequent exacerbations [5,10,11,12], where its beneficial effects on lung inflammation would be more apparent. Other effects of roflumilast, such as its augmentation of the activity of the Cl^−^ ion channel CFTR in lung epithelium, may also contribute to it benefits in these patients (see ref. [9] for a review).

There is an interesting paradox between the therapeutic actions of PDE4-selective inhibitors, such as roflumilast, in obstructive pulmonary disease, compared to the actions of β-adrenergic agonists. β-adrenergic agonists have acute bronchodilator activity [16,140,141], while roflumilast does not. Yet it is clear that both classes of agents act by increasing intracellular cAMP levels (Figure 1). Potential reasons for the differences in therapeutic effect of these two classes of agents may reflect differences in target tissues (i.e., airway smooth muscle vs. polarized pulmonary epithelium and inflammatory cells), or underlying disease (i.e., COPD with an asthmatic component vs. other sub-types of COPD), or delivery (i.e., inhaled vs. oral). Intriguingly, ensifentrine does have acute bronchodilator activity (see references above); whether this therapeutic effect is mediated through PDE4 inhibition, or instead by inhibition of PDE3, or to some other factor (e.g., inhaled vs. oral delivery) remains to be determined.

### 6.2. Lessons from Human Inflammatory Disorders That Lead to Fibrotic Lung Disease

Patients with several well-defined systemic human immune/inflammatory diseases, including scleroderma and rheumatoid arthritis, can develop interstitial lung disease (ILD), which can progress to IPF or PPF [18,53,142]. Scleroderma (synonym: systemic sclerosis) is characterized by fibrosis of the skin, musculoskeletal system, and other tissues and the development of several different antibodies. Patients with scleroderma can develop ILD that progressed to PPF; nintedanib has been shown to have benefit in pre-clinical models of scleroderma and has been shown in clinical trials to slow the progression to PPF in these patients [54,65,143]. Patients with rheumatoid arthritis, who typically present with symptoms related to the musculoskeletal system, and who develop antibodies against citrullinated proteins, can develop ILD that can progress to PPF [144]; pirfenidone has been shown to have clinical benefit in this specific patient population [145]. Germline variants in a number of genes, most notably *MUC5B* and *SETPC*, are major predisposing factors to the development of IPF in many of these disorders [142,146,147]. Patients with these disorders have shown in clinical trials to benefit from nerandomilast [18] and also from pirfenidone and nintedanib (see references above); head-to-head comparisons of these three drugs have yet to be reported. The pre-clinical models of these disorders, the results obtained from many areas of clinical investigation, and the clinical trial data are all consistent with concept that ongoing inflammation is key to fibrotic lung disease and that targeting inflammation is key to the beneficial effects of the three drugs currently available for these disorders.

### 6.3. PDE4 Inhibitors in COPD and Pulmonary Fibrosis: Different Diseases, Common Mechanisms of Action

Given that inflammation plays an essential role in the pathogenesis of both COPD and pulmonary fibrosis, and that the major therapeutic effects of both roflumilast and nerandomilast are achieved through the targeting of PDE4 isoforms in lung inflammatory cells, what exactly are the differences between these two drugs? In many of the pre-clinical models, the effects of the two drugs appear to be similar. Even their non-inflammatory effects, such as their actions on CFTR (in COPD) and their direct effects on fibroblasts (in pulmonary fibrosis), appear to be similar; indeed, these effects may help patients with both disorders. There are many residual questions: Does the broader action of roflumilast (i.e., activity against all PDE4 isoforms and therefore against an extensive range of pulmonary pathways) contribute to its therapeutic benefit? Alternatively, does the narrower action of nerandomilast (specific for PDE4B and, to some extent, PDE4D isoforms, and the pathways mediated specifically by these isoforms) a major contributor to its therapeutic benefit?

### 6.4. The Comparative “Druggability” of Roflumilast and Nerandomilast

In the design of small-molecule therapies, many factors influence the “druggability” of any individual compound, which in turn can be pivotal to its eventual success in humans. For example, solubility, protein-binding, stability in vivo, bioavailability, dosing, tissue availability, and pharmacokinetics are key factors. Such factors may have played a central role in the development of nerandomilast and therefore account for its potentially greater benefit over older drugs, such as roflumilast, in pulmonary fibrosis and potentially in other indications [148].

Related, but technically separate, from druggability is the question of tolerability (side effects) in humans. Useful tolerability data can be obtained from early-stage clinical trials, but important data may not emerge until later in the drug-testing process. The extensive phase 3 trials on both roflumilast and nerandomilast provide extensive data on the tolerability of both drugs (see references cited in the first section, above). Both agents are well-tolerated, with discontinuation percentages in the low-single digits. Diarrhea, mild nausea and fatigue are the most common side effects; alterations of mood and affect also occur, but are rarer. All these side effects are typical of PDE4-selective inhibitors as a class. We have developed preliminary data suggesting that the diarrhea seen with PDE4 inhibitors reflects their augmentation of CFTR Cl^−^ channel activity in the GI tract [138], consistent with a class effect. Current data on the relative toxicity/tolerability of roflumilast and nerandomilast needs to be interpreted with caution because of differences in patient populations in the various clinical trials reported to date. Ideally, data comparing the relatively tolerability of these two agents should be obtained by head-to-head, randomized, blinded, comparisons of both tolerability and efficacy. Therefore, a review of comparative head-to-head clinical trials of various PDE4 inhibitors, including these two drugs, in lung and other indications, would certainly be of interest.

## 7. Ongoing and Possible Future Clinical Trials of PDE4 Inhibitors and Other Agents in COPD and Pulmonary Fibrosis

### 7.1. Currently Recruiting Clinical Trials of Roflumilast, Nerandomilast, Pirfenidone and Nintedanib

To obtain a better perspective on the future roles of PDE4 inhibitors in COPD and pulmonary fibrosis, we searched www.ClinicalTrials.gov for all actively recruiting clinical trials for roflumilast, nerandomilast, pirfenidone and nintedanib in pulmonary disorders as of 28 August 2025 (see Supplemental File). This search yielded 19 clinical trials; in contrast, a search for ongoing clinical trials of all agents in COPD, performed 30 October 2023, yielded a total of 480 trials [9]. Among the most important trials identified in the current search is a phase 3 multicenter trial comparing roflumilast to the antimicrobial azithromycin as prevention of COPD exacerbations (NCT04069312) and a comparison of roflumilast to the inhaled PDE4 inhibitor tanimilast as maintenance therapy in COPD (NCT04636814; for further discussion of the pre-clinical and early-stage development of tanimilast and ensifentrine, see a prior review [9]). Also noteworthy is the PROGRESSION trial (NCT03939520), a randomized phase 3 trial comparing the efficacy and tolerance of the combination of pirfenidone and nintedanib, compared to either as monotherapy, in previously treated patients with IPF.

### 7.2. Considerations for the Design of Future Clinical Trials

Review of completed and ongoing clinical trials of PDE4-selective inhibitors in COPD and pulmonary fibrosis has shown that much progress has been made, but also that there is room for improvement in trial design. One of the key deficiencies is the lack of head-to-head comparisons of many of the available, or soon-to-be-available agents, as discussed in detail in several sections above. Several other potential areas of improvement can be identified: At what point should intervention be initiated (e.g., pre-clinical vs. symptomatic)? What biomarkers (e.g., information obtained from blood and/or tissue samples) or other procedures, such as imaging or spirometry [149,150]), can be used to determine eligibility and to stratify patients on these trials, and in clinical practice? Several recent attempts to identify and validate biomarkers in both COPD and pulmonary fibrosis are discussed in the next section. What end-points should be chosen in the design of the newer clinical trials? Greater use of biomarkers and other intermediate end-points should facilitate more rapid, and potentially smaller, clinical trials and is an important goal. Conversely, trials that focus on clinically meaningful endpoints (reductions in hospitalizations, exacerbations, and improvements in quality of life) would seem to be essential for assessing the ultimate value of newer therapies [151,152]. A related question is that of duration: given the chronicity of these diseases, how long should patients be treated on a trial before determining whether the intervention(s) are effective? In the ongoing interactions between drug-development teams, regulators, clinical trialists and patients, careful attention to these issues would seem to be essential.

## 8. Future Directions: Subclassifications, Biomarkers, and Pathways

**Figure 3:**
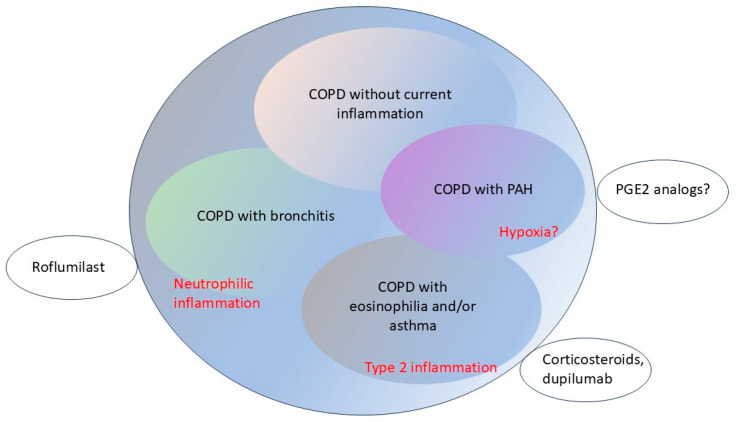
Subclasses of COPD. A tentative division of COPD into subclasses that (a) reflects underlying disease mechanisms; (b) can be used in decision-making in individual patients; (c) can be used to stratify patients on clinical trials and (d) can serve to identify new end-points in early-stage clinical trials. It includes the following subclasses: (1) Patients with predominantly manifestations of bronchitis. Neutrophil inflammation is prominent and patients may derive particular benefit from roflumilast and/or ensifentrine. (2) Patients with eosinophilia. These patients typically also have an elevated FeNO. Type 2 inflammation is predominant and patients may derive particular benefit from inhaled corticosteroids and dupilumab. (3) Patients without elevated markers of inflammation. Active inflammation is less apparent, even in the presence of profound airway obstruction. There are fewer options available specifically for these patients. (4) Patients with COPD and pulmonary arterial hypertension (PAH). These patients may derive particular benefit from inhaled or oral prostaglandin therapy, although phase 3 data are currently conflicting and/or pending. All 4 subclasses also typically benefit from inhaled muscarinic antagonists, pulmonary rehabilitation, and effective treatment of exacerbations.

Several new features are dominating the evolving landscape of novel therapies in both COPD and pulmonary fibrosis. The first, clearly, is the novel and important division of both these disorders into broad sub-classes that guide therapy and that are also useful in stratifying patients on clinical trials. In COPD, the use of eosinophilia as a marker for Type 2 inflammation has identified a subclass of patients that respond to cytokine-modulating agents, such as dupilumab and mepolizumab. In pulmonary fibrosis, the division of patients into two broad categories—IPF and PPF—has been useful in stratifying patients into appropriate clinical trials and, in the case of PPF, has the potential to base therapies on the pathobiology of the underlying disease. Current national and international guidelines are beginning to incorporate these concepts into routine clinical practice [16,21,29,31]. These stratification criteria lead to a tentative COPD classification system that reflects the underlying pathophysiology and which directly affects clinical decision-making (Figure 3, refs. [9,153,154]).

The second feature, related to the first, is the development of biomarkers that (1) reflect underlying disease mechanisms; (2) can be used in decision-making in individual patients; (3) can be used to stratify patients on clinical trials; (4) can serve as end-points in early-stage clinical trials. An emerging biomarker in COPD is an elevated fractional exhaled nitric oxide (FeNO) or alveolar NO level, which reflects ongoing inflammation, is frequently correlated with eosinophilia, and helps to identify patients with a COPD and asthma overlap phenotype [155,156,157,158,159,160,161]. Several recent publications have shown grounds for promise in identifying additional blood and tissue biomarkers, such as IL-1β, CXCL10, Eotaxin-3, PARC and IgE, in both COPD [25,39,41,162] and pulmonary fibrosis [53,61,163]. New, emerging technologies, such as single-cell sequencing on patient-derived samples (e.g., blood, sputum, BAL fluid and biopsies; ref. [164]), should contribute substantially to biomarker efforts. Additionally, there is emerging data on the use of germline genetic markers predictive of response; among the most interesting of these is the identification of polymorphisms (SNPs) in the PDE4 genes that correlate with responsiveness to the PDE4-selective inhibitor apremilast in psoriasis [165]; there is a need for similar studies in the pulmonary area.

It is apparent that study of the mechanisms of action of PDE4 drugs in a variety of human diseases, including lung disease, has enabled progress on understanding fundamental disease mechanisms in COPD and pulmonary fibrosis. Further study of these mechanisms is essential for the development of newer agents in these diseases, which would include not only PDE4 inhibitors, but agents targeting novel targets. One example is the possible use of CFTR potentiators in the treatment of COPD, which has a compelling pre-clinical rationale (reviewed in ref. [9]) and has been the subject of early-phase clinical trials [166].

Further effort, both pre-clinical and clinical, will be required to determine whether the differences postulated between various drugs in different pulmonary diseases reflects differences in the action(s) of the drugs (i.e., selectivity for certain targets), or difference in the underlying diseases (i.e., the presence of specific drug targets in different disorders), or other factors. We are in the midst of an exciting time for pulmonary therapeutics and additional insights and additional agents are emerging in a rapid fashion.

## Figures and Tables

**Table 1:** PDE4-selective inhibitors currently available for clinical use.

PDE4-Selective Inhibitor	Roflumilast	Roflumilast	Crisaborole	Ensifentrine	Nerandomilast	Apremilast
Route	Oral	Topical	Topical	Inhaled	Oral	Oral
Selectivity	Pan-PDE4	Pan-PDE4	Pan-PDE4	PDE3/4	PDE4B, PDE4D	Pan-PDE4
Clinical indications	COPD	Eczema, atopic dermatitis	Eczema, atopic dermatitis	COPD	Pulmonary fibrosis	Psoriasis, psoriatic arthritis
References	[10,11,12]	[13,14]	[15]	[16,17]	[18,19]	[20]

**Table 2:** Drugs active in pulmonary fibrosis.

Drug	Pirfenidone	Nintedanib	Nerandomilast	Treprostinil	Treprostinil
Targets	(see text)	Receptor tyrosine-kinases for VEGF, PDGF, FGF	PDE4B/D	Prostaglandin receptor	Prostaglandin receptor
Route	Oral	Oral	Oral	Inhaler	Oral
Other diseases		Scleroderma			
References	(see text)	[63,64,65,66]	[19,67]	(see text)	

**Table 3:** Targets for PDE4 inhibitors: PDE4 isoform expression and function in key pulmonary cells.

Cell/Tissue Type	Respiratory Epithelium	Ionocytes	Neutrophils	Macrophages	Fibroblasts	Eosinophils
Major pathways and mediators	Mucociliary clearance	CFTR Cl- activity	ROS, IL-8	TNFα	TGF-β	
Roflumilast target(s) in COPD	Pan-PDE4	Pan-PDE4	Pan-PDE4	Pan-PDE4	Pan-PDE4	Pan-PDE4
Nerandomilast Target(s) in IPF	(uncertain)	(uncertain)	PDE4B/D	PDE4B/D	PDE4B/D	(uncertain)

References for Table 3: For respiratory epithelium: [8,108,113,117]; for ionocytes: [9,113,117,138,139]; for neutrophils: [8,95,108,113,117]; for macrophages: [8,54,95,108,113,117]; for fibroblasts: [8,108,113,117,118,119,120,121,123,124,125,126,127,128,133,136,137]; for eosinophils: [8,95,108,113,117].

## Data Availability

No new data were created or analyzed in this study. Data sharing is not applicable to this article.

## Supplementary Materials

The following supporting information can be downloaded at: https://www.mdpi.com/article/10.3390/life15101600/s1, Trials at ClinicalTrials.gov (xlxs).

## References

[1] Kelly M.P., Nikolaev V.O., Gobejishvili L., Lugnier C., Hesslinger C., Nickolaus P., Kass D.A., Pereira de Vasconcelos W., Fischmeister R., Brocke S. (2025). Cyclic nucleotide phosphodiesterases as drug targets. Pharmacol. Rev..

[2] Hanania N.A., Celli B.R. (2025). Phosphodiesterase Inhibition as a Therapeutic Strategy for Chronic Obstructive Pulmonary Disease: Where We Have Been and What Lies Ahead. Chronic Obstr. Pulm. Dis..

[3] Conti M., Beavo J. (2007). Biochemistry and physiology of cyclic nucleotide phosphodiesterases: Essential components in cyclic nucleotide signaling. Annu. Rev. Biochem..

[4] Baillie G.S., Tejeda G.S., Kelly M.P. (2019). Therapeutic targeting of 3′,5′-cyclic nucleotide phosphodiesterases: Inhibition and beyond. Nat. Rev. Drug Discov..

[5] Janjua S., Fortescue R., Poole P. (2020). Phosphodiesterase-4 inhibitors for chronic obstructive pulmonary disease. Cochrane Database Syst. Rev..

[6] Francis S.H., Blount M.A., Corbin J.D. (2011). Mammalian cyclic nucleotide phosphodiesterases: Molecular mechanisms and physiological functions. Physiol. Rev..

[7] Maurice D.H., Ke H., Ahmad F., Wang Y., Chung J., Manganiello V.C. (2014). Advances in targeting cyclic nucleotide phosphodiesterases. Nat. Rev. Drug Discov..

[8] Hatzelmann A., Morcillo E.J., Lungarella G., Adnot S., Sanjar S., Beume R., Schudt C., Tenor H. (2010). The preclinical pharmacology of roflumilast—A selective, oral phosphodiesterase 4 inhibitor in development for chronic obstructive pulmonary disease. Pulm. Pharmacol. Ther..

[9] Bolger G.B. (2023). Therapeutic Targets and Precision Medicine in COPD: Inflammation, Ion Channels, Both, or Neither?. Int. J. Mol. Sci..

[10] Calverley P.M., Rabe K.F., Goehring U.M., Kristiansen S., Fabbri L.M., Martinez F.J. (2009). Roflumilast in symptomatic chronic obstructive pulmonary disease: Two randomised clinical trials. Lancet.

[11] Fabbri L.M., Calverley P.M., Izquierdo-Alonso J.L., Bundschuh D.S., Brose M., Martinez F.J., Rabe K.F. (2009). Roflumilast in moderate-to-severe chronic obstructive pulmonary disease treated with longacting bronchodilators: Two randomised clinical trials. Lancet.

[12] Martinez F.J., Calverley P.M., Goehring U.M., Brose M., Fabbri L.M., Rabe K.F. (2015). Effect of roflumilast on exacerbations in patients with severe chronic obstructive pulmonary disease uncontrolled by combination therapy (REACT): A multicentre randomised controlled trial. Lancet.

[13] (2024). Roflumilast foam (Zoryve) for seborrheic dermatitis. Med. Lett. Drugs Ther..

[14] (2024). Roflumilast cream (Zoryve) for atopic dermatitis. Med. Lett. Drugs Ther..

[15] (2017). Crisaborole (Eucrisa) for atopic dermatitis. Med. Lett. Drugs Ther..

[16] (2024). Drugs for COPD. Med. Lett. Drugs Ther..

[17] (2024). Ensifentrine (Ohtuvayre) for COPD. Med. Lett. Drugs Ther..

[18] Maher T.M., Assassi S., Azuma A., Cottin V., Hoffmann-Vold A.M., Kreuter M., Oldham J.M., Richeldi L., Valenzuela C., Wijsenbeek M.S. (2025). Nerandomilast in Patients with Progressive Pulmonary Fibrosis. N. Engl. J. Med..

[19] Richeldi L., Azuma A., Cottin V., Kreuter M., Maher T.M., Martinez F.J., Oldham J.M., Valenzuela C., Clerisme-Beaty E., Gordat M. (2025). Nerandomilast in Patients with Idiopathic Pulmonary Fibrosis. N. Engl. J. Med..

[20] (2024). Drugs for plaque psoriasis. Med. Lett. Drugs Ther..

[21] (2025). Global Initiative for Chronic Obstructive Lung Disease: 2025 Report. https://goldcopd.org/2025-gold-report/.

[22] Hogg J.C., Timens W. (2009). The pathology of chronic obstructive pulmonary disease. Annu. Rev. Pathol..

[23] Stuart-Harris C.H. (1965). Definition and classification of chronic bronchitis for clinical and epidemiological purposes. A report to the Medical Research Council by their Committee on the Aetiology of Chronic Bronchitis. Lancet.

[24] Pizzichini E., Pizzichini M.M., Gibson P., Parameswaran K., Gleich G.J., Berman L., Dolovich J., Hargreave F.E. (1998). Sputum eosinophilia predicts benefit from prednisone in smokers with chronic obstructive bronchitis. Am. J. Respir. Crit. Care Med..

[25] Bafadhel M., McKenna S., Terry S., Mistry V., Reid C., Haldar P., McCormick M., Haldar K., Kebadze T., Duvoix A. (2011). Acute exacerbations of chronic obstructive pulmonary disease: Identification of biologic clusters and their biomarkers. Am. J. Respir. Crit. Care Med..

[26] Brightling C.E., Monteiro W., Ward R., Parker D., Morgan M.D., Wardlaw A.J., Pavord I.D. (2000). Sputum eosinophilia and short-term response to prednisolone in chronic obstructive pulmonary disease: A randomised controlled trial. Lancet.

[27] Siva R., Green R.H., Brightling C.E., Shelley M., Hargadon B., McKenna S., Monteiro W., Berry M., Parker D., Wardlaw A.J. (2007). Eosinophilic airway inflammation and exacerbations of COPD: A randomised controlled trial. Eur. Respir. J..

[28] Pascoe S., Locantore N., Dransfield M.T., Barnes N.C., Pavord I.D. (2015). Blood eosinophil counts, exacerbations, and response to the addition of inhaled fluticasone furoate to vilanterol in patients with chronic obstructive pulmonary disease: A secondary analysis of data from two parallel randomised controlled trials. Lancet Respir. Med..

[29] Singh D., Agusti A., Martinez F.J., Papi A., Pavord I.D., Wedzicha J.A., Vogelmeier C.F., Halpin D.M.G. (2022). Blood Eosinophils and Chronic Obstructive Pulmonary Disease: A Global Initiative for Chronic Obstructive Lung Disease Science Committee 2022 Review. Am. J. Respir. Crit. Care Med..

[30] (2020). Drugs for COPD. Med. Lett. Drugs Ther..

[31] Agusti A., Celli B.R., Criner G.J., Halpin D., Anzueto A., Barnes P., Bourbeau J., Han M.K., Martinez F.J., Montes de Oca M. (2023). Global Initiative for Chronic Obstructive Lung Disease 2023 Report: GOLD Executive Summary. Am. J. Respir. Crit. Care Med..

[32] Rennard S.I., Calverley P.M., Goehring U.M., Bredenbroker D., Martinez F.J. (2011). Reduction of exacerbations by the PDE4 inhibitor roflumilast—The importance of defining different subsets of patients with COPD. Respir. Res..

[33] Anzueto A., Barjaktarevic I.Z., Siler T.M., Rheault T., Bengtsson T., Rickard K., Sciurba F. (2023). Ensifentrine, a Novel Phosphodiesterase 3 and 4 Inhibitor for the Treatment of Chronic Obstructive Pulmonary Disease: Randomized, Double-Blind, Placebo-controlled, Multicenter Phase III Trials (the ENHANCE Trials). Am. J. Respir. Crit. Care Med..

[34] Sciurba F.C., Christenson S.A., Rheault T., Bengtsson T., Rickard K., Barjaktarevic I.Z. (2025). Effect of Dual Phosphodiesterase 3 and 4 Inhibitor Ensifentrine on Exacerbation Rate and Risk in Patients with Moderate to Severe COPD. Chest.

[35] Yappalparvi A., Balaraman A.K., Padmapriya G., Gaidhane S., Kaur I., Lal M., Iqbal S., Prasad G.V.S., Pramanik A., Vishwakarma T. (2025). Safety and efficacy of ensifentrine in COPD: A systemic review and meta-analysis. Respir. Med..

[36] Hammadeh B.M., Younis O.M., Alsufi M.I., Idrees M., Hussein A.M., Aldalati A.Y., Qtaishat F.A., Qatawneh B., Bugazia A., Hamed R.A. (2025). Efficacy and safety of ensifentrine in treatment of COPD: A systematic review and meta-analysis of clinical trials. Ther. Adv. Respir. Dis..

[37] Calzetta L., Cazzola M., Gholamalishahi S., Rogliani P. (2024). The novel inhaled dual PDE3 and PDE4 inhibitor ensifentrine for the treatment of COPD: A systematic review and meta-analysis protocol on trough FEV(1) and exacerbation according to PRISMA statement. Curr. Res. Pharmacol. Drug Discov..

[38] Boswell-Smith V., Spina D., Oxford A.W., Comer M.B., Seeds E.A., Page C.P. (2006). The pharmacology of two novel long-acting phosphodiesterase 3/4 inhibitors, RPL554 [9,10-dimethoxy-2(2,4,6-trimethylphenylimino)-3-(n-carbamoyl-2-aminoethyl)-3,4,6, 7-tetrahydro-2H-pyrimido [6,1-a]isoquinolin-4-one] and RPL565 [6,7-dihydro-2-(2,6-diisopropylphenoxy)-9,10-dimethoxy-4H-pyrimido[6,1-a]isoquino lin-4-one]. J. Pharmacol. Exp. Ther..

[39] Bhatt S.P., Rabe K.F., Hanania N.A., Vogelmeier C.F., Cole J., Bafadhel M., Christenson S.A., Papi A., Singh D., Laws E. (2023). Dupilumab for COPD with Type 2 Inflammation Indicated by Eosinophil Counts. N. Engl. J. Med..

[40] Le Floc’h A., Allinne J., Nagashima K., Scott G., Birchard D., Asrat S., Bai Y., Lim W.K., Martin J., Huang T. (2020). Dual blockade of IL-4 and IL-13 with dupilumab, an IL-4Ralpha antibody, is required to broadly inhibit type 2 inflammation. Allergy.

[41] Bhatt S.P., Rabe K.F., Hanania N.A., Vogelmeier C.F., Bafadhel M., Christenson S.A., Papi A., Singh D., Laws E., Patel N. (2024). Dupilumab for COPD with Blood Eosinophil Evidence of Type 2 Inflammation. N. Engl. J. Med..

[42] Pavord I.D., Chanez P., Criner G.J., Kerstjens H.A.M., Korn S., Lugogo N., Martinot J.B., Sagara H., Albers F.C., Bradford E.S. (2017). Mepolizumab for Eosinophilic Chronic Obstructive Pulmonary Disease. N. Engl. J. Med..

[43] Sciurba F.C., Criner G.J., Christenson S.A., Martinez F.J., Papi A., Roche N., Bourbeau J., Korn S., Bafadhel M., Han M.K. (2025). Mepolizumab to Prevent Exacerbations of COPD with an Eosinophilic Phenotype. N. Engl. J. Med..

[44] Li S., Yi B., Wang H., Xu X., Yu L. (2025). Efficacy and Safety of Biologics Targeting Type 2 Inflammation in COPD: A Systematic Review and Network Meta-Analysis. Int. J. Chronic Obstr. Pulm. Dis..

[45] Mohamed M.M.G., Kamel G., Charbek E. (2025). Role of Monoclonal Antibodies in the Management of Eosinophilic Chronic Obstructive Pulmonary Disease: A Meta-analysis of Randomized Controlled Trials. Ann. Am. Thorac. Soc..

[46] Nelms K., Keegan A.D., Zamorano J., Ryan J.J., Paul W.E. (1999). The IL-4 receptor: Signaling mechanisms and biologic functions. Annu. Rev. Immunol..

[47] Hurdayal R., Brombacher F. (2017). Interleukin-4 Receptor Alpha: From Innate to Adaptive Immunity in Murine Models of Cutaneous Leishmaniasis. Front. Immunol..

[48] (2025). Mepolizumab (Nucala) for COPD. Med. Lett. Drugs Ther..

[49] Mosmann T.R., Cherwinski H., Bond M.W., Giedlin M.A., Coffman R.L. (1986). Two types of murine helper T cell clone. I. Definition according to profiles of lymphokine activities and secreted proteins. J. Immunol..

[50] Spellberg B., Edwards J.E. (2001). Type 1/Type 2 immunity in infectious diseases. Clin. Infect. Dis..

[51] Fahy J.V. (2015). Type 2 inflammation in asthma–present in most, absent in many. Nat. Rev. Immunol..

[52] Raghu G., Remy-Jardin M., Richeldi L., Thomson C.C., Inoue Y., Johkoh T., Kreuter M., Lynch D.A., Maher T.M., Martinez F.J. (2022). Idiopathic Pulmonary Fibrosis (an Update) and Progressive Pulmonary Fibrosis in Adults: An Official ATS/ERS/JRS/ALAT Clinical Practice Guideline. Am. J. Respir. Crit. Care Med..

[53] Maher T.M. (2024). Interstitial Lung Disease: A Review. JAMA.

[54] Baas J.D., Varga J., Feghali-Bostwick C., Peters-Golden M., Fortier S.M. (2025). Distinct cAMP Regulation in Scleroderma Lung and Skin Myofibroblasts Governs Their Dedifferentiation via p38alpha Inhibition. FASEB J..

[55] Conte P., Ascierto P.A., Patelli G., Danesi R., Vanzulli A., Sandomenico F., Tarsia P., Cattelan A., Comes A., De Laurentiis M. (2022). Drug-induced interstitial lung disease during cancer therapies: Expert opinion on diagnosis and treatment. ESMO Open.

[56] Mushiroda T., Wattanapokayakit S., Takahashi A., Nukiwa T., Kudoh S., Ogura T., Taniguchi H., Kubo M., Kamatani N., Nakamura Y. (2008). A genome-wide association study identifies an association of a common variant in TERT with susceptibility to idiopathic pulmonary fibrosis. J. Med. Genet..

[57] Borie R., Tabeze L., Thabut G., Nunes H., Cottin V., Marchand-Adam S., Prevot G., Tazi A., Cadranel J., Mal H. (2016). Prevalence and characteristics of TERT and TERC mutations in suspected genetic pulmonary fibrosis. Eur. Respir. J..

[58] Dressen A., Abbas A.R., Cabanski C., Reeder J., Ramalingam T.R., Neighbors M., Bhangale T.R., Brauer M.J., Hunkapiller J., Reeder J. (2018). Analysis of protein-altering variants in telomerase genes and their association with MUC5B common variant status in patients with idiopathic pulmonary fibrosis: A candidate gene sequencing study. Lancet Respir. Med..

[59] Peljto A.L., Blumhagen R.Z., Walts A.D., Cardwell J., Powers J., Corte T.J., Dickinson J.L., Glaspole I., Moodley Y.P., Vasakova M.K. (2023). Idiopathic Pulmonary Fibrosis Is Associated with Common Genetic Variants and Limited Rare Variants. Am. J. Respir. Crit. Care Med..

[60] Miedema J.R., Moor C.C., Veltkamp M., Baart S., Lie N.S.L., Grutters J.C., Wijsenbeek M.S., Mostard R.L.M. (2022). Safety and tolerability of pirfenidone in asbestosis: A prospective multicenter study. Respir. Res..

[61] Chang S., Wen S., Zhang W., Zhang H., Guo Y., Wang Q., Hu X., Liu Z., Sun Y., Yang A. (2025). The updated evidence of pirfenidone treated silicosis based on network pharmacology, molecular docking and experimental validation. Front. Med..

[62] Behr J. (2013). The diagnosis and treatment of idiopathic pulmonary fibrosis. Dtsch. Arztebl. Int..

[63] Richeldi L., Costabel U., Selman M., Kim D.S., Hansell D.M., Nicholson A.G., Brown K.K., Flaherty K.R., Noble P.W., Raghu G. (2011). Efficacy of a tyrosine kinase inhibitor in idiopathic pulmonary fibrosis. N. Engl. J. Med..

[64] Richeldi L., du Bois R.M., Raghu G., Azuma A., Brown K.K., Costabel U., Cottin V., Flaherty K.R., Hansell D.M., Inoue Y. (2014). Efficacy and safety of nintedanib in idiopathic pulmonary fibrosis. N. Engl. J. Med..

[65] Distler O., Highland K.B., Gahlemann M., Azuma A., Fischer A., Mayes M.D., Raghu G., Sauter W., Girard M., Alves M. (2019). Nintedanib for Systemic Sclerosis-Associated Interstitial Lung Disease. N. Engl. J. Med..

[66] Flaherty K.R., Wells A.U., Cottin V., Devaraj A., Walsh S.L.F., Inoue Y., Richeldi L., Kolb M., Tetzlaff K., Stowasser S. (2019). Nintedanib in Progressive Fibrosing Interstitial Lung Diseases. N. Engl. J. Med..

[67] Richeldi L., Azuma A., Cottin V., Hesslinger C., Stowasser S., Valenzuela C., Wijsenbeek M.S., Zoz D.F., Voss F., Maher T.M. (2022). Trial of a Preferential Phosphodiesterase 4B Inhibitor for Idiopathic Pulmonary Fibrosis. N. Engl. J. Med..

[68] King T.E., Bradford W.Z., Castro-Bernardini S., Fagan E.A., Glaspole I., Glassberg M.K., Gorina E., Hopkins P.M., Kardatzke D., Lancaster L. (2014). A phase 3 trial of pirfenidone in patients with idiopathic pulmonary fibrosis. N. Engl. J. Med..

[69] Behr J., Bendstrup E., Crestani B., Gunther A., Olschewski H., Skold C.M., Wells A., Wuyts W., Koschel D., Kreuter M. (2016). Safety and tolerability of acetylcysteine and pirfenidone combination therapy in idiopathic pulmonary fibrosis: A randomised, double-blind, placebo-controlled, phase 2 trial. Lancet Respir. Med..

[70] Noble P.W., Albera C., Bradford W.Z., Costabel U., du Bois R.M., Fagan E.A., Fishman R.S., Glaspole I., Glassberg M.K., Lancaster L. (2016). Pirfenidone for idiopathic pulmonary fibrosis: Analysis of pooled data from three multinational phase 3 trials. Eur. Respir. J..

[71] Maher T.M., Corte T.J., Fischer A., Kreuter M., Lederer D.J., Molina-Molina M., Axmann J., Kirchgaessler K.U., Samara K., Gilberg F. (2020). Pirfenidone in patients with unclassifiable progressive fibrosing interstitial lung disease: A double-blind, randomised, placebo-controlled, phase 2 trial. Lancet Respir. Med..

[72] Vancheri C., Kreuter M., Richeldi L., Ryerson C.J., Valeyre D., Grutters J.C., Wiebe S., Stansen W., Quaresma M., Stowasser S. (2018). Nintedanib with Add-on Pirfenidone in Idiopathic Pulmonary Fibrosis. Results of the INJOURNEY Trial. Am. J. Respir. Crit. Care Med..

[73] Ikeda S., Sekine A., Baba T., Kato T., Katano T., Tabata E., Shintani R., Yamakawa H., Oda T., Okuda R. (2022). Randomized phase II study of nintedanib with or without pirfenidone in patients with idiopathic pulmonary fibrosis who experienced disease progression during prior pirfenidone administration. Medicine.

[74] Qiu Y., Ye W. (2025). Therapeutic efficacy of pirfenidone and nintedanib in pulmonary fibrosis; a systematic review and meta-analysis. Ann. Thorac. Med..

[75] Di Martino E., Provenzani A., Vitulo P., Polidori P. (2021). Systematic Review and Meta-analysis of Pirfenidone, Nintedanib, and Pamrevlumab for the Treatment of Idiopathic Pulmonary Fibrosis. Ann. Pharmacother..

[76] Lee J.S. (2025). Progress through Persistence-Turning the Page in Pulmonary Fibrosis Clinical Trials. N. Engl. J. Med..

[77] Sgalla G., Simonetti J., Cortese S., Richeldi L. (2023). BI 1015550: An investigational phosphodiesterase 4B (PDE4B) inhibitor for lung function decline in idiopathic pulmonary fibrosis (IPF). Expert Opin. Investig. Drugs.

[78] Nathan S.D., Behr J., Cottin V., Lancaster L., Smith P., Deng C.Q., Pearce N., Bell H., Peterson L., Flaherty K.R. (2022). Study design and rationale for the TETON phase 3, randomised, controlled clinical trials of inhaled treprostinil in the treatment of idiopathic pulmonary fibrosis. BMJ Open Respir. Res..

[79] Ismat F.A., Usansky H.H., Villa R., Zou J., Teper A. (2022). Safety, Tolerability, and Pharmacokinetics of Treprostinil Palmitil Inhalation Powder for Pulmonary Hypertension: A Phase 1, Randomized, Double-Blind, Single- and Multiple-Dose Study. Adv. Ther..

[80] Cullivan S., Genecand L., El-Merhie N., MacKenzie A., Lichtblau M. (2025). Inhaled treprostinil in group 3 pulmonary hypertension associated with lung disease: Results of the INCREASE and PERFECT studies. Breathe.

[81] Nathan S.D., Johri S., Joly J.M., King C.S., Raina A., McEvoy C.A., Lee D., Shen E., Smith P., Deng C. (2024). Survival analysis from the INCREASE study in PH-ILD: Evaluating the impact of treatment crossover on overall mortality. Thorax.

[82] Sahay S., Palevsky H., El-Kersh K., Restrepo-Jaramillo R., Bajwa A.A., Desai S., Joly J.M., Spikes L.A., Eggert M.S., Johri S. (2025). BREEZE Optional Extension Phase: Long-term safety and efficacy of treprostinil dry powder inhaler (Tyvaso DPI) in pulmonary arterial hypertension. Respir. Med..

[83] Bolger G.B. (2021). The PDE-Opathies: Diverse Phenotypes Produced by a Functionally Related Multigene Family. Trends Genet..

[84] Gloerich M., Bos J.L. (2010). Epac: Defining a new mechanism for cAMP action. Annu. Rev. Pharmacol. Toxicol..

[85] Schmidt M., Dekker F.J., Maarsingh H. (2013). Exchange protein directly activated by cAMP (epac): A multidomain cAMP mediator in the regulation of diverse biological functions. Pharmacol. Rev..

[86] Santoro B., Shah M.M. (2020). Hyperpolarization-Activated Cyclic Nucleotide-Gated Channels as Drug Targets for Neurological Disorders. Annu. Rev. Pharmacol. Toxicol..

[87] Tucker S.J., Zorn A.J. (2022). The role of Popeye domain-containing protein 1 (POPDC1) in the progression of the malignant phenotype. Br. J. Pharmacol..

[88] Tibbo A.J., Mika D., Dobi S., Ling J., McFall A., Tejeda G.S., Blair C., MacLeod R., MacQuaide N., Gok C. (2022). Phosphodiesterase type 4 anchoring regulates cAMP signaling to Popeye domain-containing proteins. J. Mol. Cell Cardiol..

[89] Smith F.D., Esseltine J.L., Nygren P.J., Veesler D., Byrne D.P., Vonderach M., Strashnov I., Eyers C.E., Eyers P.A., Langeberg L.K. (2017). Local protein kinase A action proceeds through intact holoenzymes. Science.

[90] Omar M.H., Scott J.D. (2020). AKAP Signaling Islands: Venues for Precision Pharmacology. Trends Pharmacol. Sci..

[91] Bucko P.J., Scott J.D. (2021). Drugs That Regulate Local Cell Signaling: AKAP Targeting as a Therapeutic Option. Annu. Rev. Pharmacol. Toxicol..

[92] Zhang K.Y., Card G.L., Suzuki Y., Artis D.R., Fong D., Gillette S., Hsieh D., Neiman J., West B.L., Zhang C. (2004). A glutamine switch mechanism for nucleotide selectivity by phosphodiesterases. Mol. Cell.

[93] Salter E.A., Wierzbicki A. (2007). The mechanism of cyclic nucleotide hydrolysis in the phosphodiesterase catalytic site. J. Phys. Chem. B.

[94] Bolger G., Michaeli T., Martins T., St J.T., Steiner B., Rodgers L., Riggs M., Wigler M., Ferguson K. (1993). A family of human phosphodiesterases homologous to the dunce learning and memory gene product of Drosophila melanogaster are potential targets for antidepressant drugs. Mol. Cell. Biol..

[95] Houslay M.D., Sullivan M., Bolger G.B. (1998). The multienzyme PDE4 cyclic AMP-specific phosphodiesterase family: Intracellular targeting, regulation, and selective inhibition by compounds exerting anti-inflammatory and anti-depressant actions. Adv. Pharmacol..

[96] Campbell S.L., van Groen T., Kadish I., Smoot L.H.M., Bolger G.B. (2017). Altered phosphorylation, electrophysiology, and behavior on attenuation of PDE4B action in hippocampus. BMC Neurosci..

[97] Bolger G.B., Smoot L.H.M., van Groen T. (2020). Dominant-Negative Attenuation of cAMP-Selective Phosphodiesterase PDE4D Action Affects Learning and Behavior. Int. J. Mol. Sci..

[98] Cedervall P., Aulabaugh A., Geoghegan K.F., McLellan T.J., Pandit J. (2015). Engineered stabilization and structural analysis of the autoinhibited conformation of PDE4. Proc. Natl. Acad. Sci. USA.

[99] Millar J.K., Pickard B.S., Mackie S., James R., Christie S., Buchanan S.R., Malloy M.P., Chubb J.E., Huston E., Baillie G.S. (2005). DISC1 and PDE4B are interacting genetic factors in schizophrenia that regulate cAMP signaling. Science.

[100] Yarwood S.J., Steele M.R., Scotland G., Houslay M.D., Bolger G.B. (1999). The RACK1 signaling scaffold protein selectively interacts with the cAMP-specific phosphodiesterase PDE4D5 isoform. J. Biol. Chem..

[101] Perry S.J., Baillie G.S., Kohout T.A., McPhee I., Magiera M.M., Ang K.L., Miller W.E., McLean A.J., Conti M., Houslay M.D. (2002). Targeting of cyclic AMP degradation to beta 2-adrenergic receptors by beta-arrestins. Science.

[102] Dodge-Kafka K.L., Soughayer J., Pare G.C., Carlisle Michel J.J., Langeberg L.K., Kapiloff M.S., Scott J.D. (2005). The protein kinase A anchoring protein mAKAP coordinates two integrated cAMP effector pathways. Nature.

[103] Sette C., Conti M. (1996). Phosphorylation and activation of a cAMP-specific phosphodiesterase by the cAMP-dependent protein kinase. Involvement of serine 54 in the enzyme activation. J. Biol. Chem..

[104] Hoffmann R., Wilkinson I.R., McCallum J.F., Engels P., Houslay M.D. (1998). cAMP-specific phosphodiesterase HSPDE4D3 mutants which mimic activation and changes in rolipram inhibition triggered by protein kinase A phosphorylation of Ser-54: Generation of a molecular model. Biochem. J..

[105] Linglart A., Fryssira H., Hiort O., Holterhus P.M., de Perez N.G., Argente J., Heinrichs C., Kuechler A., Mantovani G., Leheup B. (2012). PRKAR1A and PDE4D Mutations Cause Acrodysostosis but Two Distinct Syndromes with or without GPCR-Signaling Hormone Resistance. J. Clin. Endocrinol. Metab..

[106] Hoffmann R., Baillie G.S., MacKenzie S.J., Yarwood S.J., Houslay M.D. (1999). The MAP kinase ERK2 inhibits the cyclic AMP-specific phosphodiesterase HSPDE4D3 by phosphorylating it at Ser579. EMBO J..

[107] Hussain M., Tejeda G.S., Baillie G.S. (2025). Posttranslational modifications of phosphodiesterase type 4 enzymes represent novel points for therapeutic targeting. FEBS J..

[108] Herrmann F.E., Hesslinger C., Wollin L., Nickolaus P. (2022). BI 1015550 is a PDE4B Inhibitor and a Clinical Drug Candidate for the Oral Treatment of Idiopathic Pulmonary Fibrosis. Front. Pharmacol..

[109] Bolger G.B., Erdogan S., Jones R.E., Loughney K., Scotland G., Hoffmann R., Wilkinson I., Farrell C., Houslay M.D. (1997). Characterization of five different proteins produced by alternatively spliced mRNAs from the human cAMP-specific phosphodiesterase PDE4D gene. Biochem. J..

[110] Travaglini K.J., Nabhan A.N., Penland L., Sinha R., Gillich A., Sit R.V., Chang S., Conley S.D., Mori Y., Seita J. (2020). A molecular cell atlas of the human lung from single-cell RNA sequencing. Nature.

[111] Montoro D.T., Haber A.L., Biton M., Vinarsky V., Lin B., Birket S.E., Yuan F., Chen S., Leung H.M., Villoria J. (2018). A revised airway epithelial hierarchy includes CFTR-expressing ionocytes. Nature.

[112] Plasschaert L.W., Zilionis R., Choo-Wing R., Savova V., Knehr J., Roma G., Klein A.M., Jaffe A.B. (2018). A single-cell atlas of the airway epithelium reveals the CFTR-rich pulmonary ionocyte. Nature.

[113] Wollin L., Bundschuh D.S., Wohlsen A., Marx D., Beume R. (2006). Inhibition of airway hyperresponsiveness and pulmonary inflammation by roflumilast and other PDE4 inhibitors. Pulm. Pharmacol. Ther..

[114] Bozyk P.D., Moore B.B. (2011). Prostaglandin E2 and the pathogenesis of pulmonary fibrosis. Am. J. Respir. Cell Mol. Biol..

[115] Wendell S.G., Fan H., Zhang C. (2020). G Protein-Coupled Receptors in Asthma Therapy: Pharmacology and Drug Action. Pharmacol. Rev..

[116] Cebulla D., van Geffen C., Kolahian S. (2023). The role of PGE2 and EP receptors on lung’s immune and structural cells; possibilities for future asthma therapy. Pharmacol. Ther..

[117] Fang Q., Ma Y., Wang J., Michalski J., Rennard S.I., Liu X. (2013). PGE 2 desensitizes beta -agonist effect on human lung fibroblast-mediated collagen gel contraction through upregulating PDE4. Mediat. Inflamm..

[118] Kohyama T., Liu X., Wen F.Q., Zhu Y.K., Wang H., Kim H.J., Takizawa H., Cieslinski L.B., Barnette M.S., Rennard S.I. (2002). PDE4 inhibitors attenuate fibroblast chemotaxis and contraction of native collagen gels. Am. J. Respir. Cell Mol. Biol..

[119] Ikari J., Michalski J.M., Iwasawa S., Gunji Y., Nogel S., Park J.H., Nelson A.J., Farid M., Wang X., Schulte N. (2013). Phosphodiesterase 4 Inhibition Augments Human Lung Fibroblast VEGF Production Induced by PGE2. Am. J. Respir. Cell Mol. Biol..

[120] Moshkovitz N., Epstein Shochet G., Shitrit D. (2023). Prostaglandin E2 (PGE2) and Roflumilast Involvement in IPF Progression. Int. J. Mol. Sci..

[121] Kolosionek E., Savai R., Ghofrani H.A., Weissmann N., Guenther A., Grimminger F., Seeger W., Banat G.A., Schermuly R.T., Pullamsetti S.S. (2009). Expression and activity of phosphodiesterase isoforms during epithelial mesenchymal transition: The role of phosphodiesterase 4. Mol. Biol. Cell.

[122] Kikuchi K., Kadono T., Ihn H., Sato S., Igarashi A., Nakagawa H., Tamaki K., Takehara K. (1995). Growth regulation in scleroderma fibroblasts: Increased response to transforming growth factor-beta 1. J. Investig. Dermatol..

[123] Togo S., Liu X., Wang X., Sugiura H., Kamio K., Kawasaki S., Kobayashi T., Ertl R.F., Ahn Y., Holz O. (2009). PDE4 inhibitors roflumilast and rolipram augment PGE2 inhibition of TGF-beta1-stimulated fibroblasts. Am. J. Physiol. Lung Cell Mol. Physiol..

[124] Wojcik-Pszczola K., Chlon-Rzepa G., Jankowska A., Slusarczyk M., Ferdek P.E., Kusiak A.A., Swierczek A., Pociecha K., Koczurkiewicz-Adamczyk P., Wyska E. (2020). A Novel, Pan-PDE Inhibitor Exerts Anti-Fibrotic Effects in Human Lung Fibroblasts via Inhibition of TGF-beta Signaling and Activation of cAMP/PKA Signaling. Int. J. Mol. Sci..

[125] Sachs B.D., Baillie G.S., McCall J.R., Passino M.A., Schachtrup C., Wallace D.A., Dunlop A.J., MacKenzie K.F., Klussmann E., Lynch M.J. (2007). p75 neurotrophin receptor regulates tissue fibrosis through inhibition of plasminogen activation via a PDE4/cAMP/PKA pathway. J. Cell Biol..

[126] Sabatini F., Petecchia L., Boero S., Silvestri M., Klar J., Tenor H., Beume R., Hatzelmann A., Rossi G.A. (2010). A phosphodiesterase 4 inhibitor, roflumilast N-oxide, inhibits human lung fibroblast functions in vitro. Pulm. Pharmacol. Ther..

[127] Tannheimer S.L., Wright C.D., Salmon M. (2012). Combination of roflumilast with a beta-2 adrenergic receptor agonist inhibits proinflammatory and profibrotic mediator release from human lung fibroblasts. Respir. Res..

[128] Cortijo J., Iranzo A., Milara X., Mata M., Cerda-Nicolas M., Ruiz-Sauri A., Tenor H., Hatzelmann A., Morcillo E.J. (2009). Roflumilast, a phosphodiesterase 4 inhibitor, alleviates bleomycin-induced lung injury. Br. J. Pharmacol..

[129] Iyer S.N., Gurujeyalakshmi G., Giri S.N. (1999). Effects of pirfenidone on transforming growth factor-beta gene expression at the transcriptional level in bleomycin hamster model of lung fibrosis. J. Pharmacol. Exp. Ther..

[130] Iyer S.N., Gurujeyalakshmi G., Giri S.N. (1999). Effects of pirfenidone on procollagen gene expression at the transcriptional level in bleomycin hamster model of lung fibrosis. J. Pharmacol. Exp. Ther..

[131] Chen W.C., Chen N.J., Chen H.P., Yu W.K., Su V.Y., Chen H., Wu H.H., Yang K.Y. (2020). Nintedanib Reduces Neutrophil Chemotaxis via Activating GRK2 in Bleomycin-Induced Pulmonary Fibrosis. Int. J. Mol. Sci..

[132] Yu W.K., Chen W.C., Su V.Y., Shen H.C., Wu H.H., Chen H., Yang K.Y. (2022). Nintedanib Inhibits Endothelial Mesenchymal Transition in Bleomycin-Induced Pulmonary Fibrosis via Focal Adhesion Kinase Activity Reduction. Int. J. Mol. Sci..

[133] Pan L., Cheng Y., Yang W., Wu X., Zhu H., Hu M., Zhang Y., Zhang M. (2023). Nintedanib Ameliorates Bleomycin-Induced Pulmonary Fibrosis, Inflammation, Apoptosis, and Oxidative Stress by Modulating PI3K/Akt/mTOR Pathway in Mice. Inflammation.

[134] Yang Y., Wang X., Zhang J. (2024). Pirfenidone and nintedanib attenuate pulmonary fibrosis in mice by inhibiting the expression of JAK2. J. Thorac. Dis..

[135] Zhong Z., Gao Y., He C., Li W., Sang L., Huang Y., Chen X., Xie M., Zhang C., Yu Y. (2025). Nintedanib improves bleomycin-induced pulmonary fibrosis by inhibiting the Clec7a/SPP1 pathway in interstitial macrophages. Cell. Signal..

[136] Udalov S., Dumitrascu R., Pullamsetti S.S., Al-tamari H.M., Weissmann N., Ghofrani H.A., Guenther A., Voswinckel R., Seeger W., Grimminger F. (2010). Effects of phosphodiesterase 4 inhibition on bleomycin-induced pulmonary fibrosis in mice. BMC Pulm. Med..

[137] Milara J., Morcillo E., Monleon D., Tenor H., Cortijo J. (2015). Roflumilast Prevents the Metabolic Effects of Bleomycin-Induced Fibrosis in a Murine Model. PLoS ONE.

[138] Lambert J.A., Raju S.V., Tang L.P., McNicholas C.M., Li Y., Courville C.A., Farris R.F., Coricor G.E., Smoot L.H., Mazur M.M. (2014). Cystic fibrosis transmembrane conductance regulator activation by roflumilast contributes to therapeutic benefit in chronic bronchitis. Am. J. Respir. Cell Mol. Biol..

[139] Blanchard E., Zlock L., Lao A., Mika D., Namkung W., Xie M., Scheitrum C., Gruenert D.C., Verkman A.S., Finkbeiner W.E. (2014). Anchored PDE4 regulates chloride conductance in wild-type and DeltaF508-CFTR human airway epithelia. FASEB J..

[140] Mosnaim G. (2023). Asthma in Adults. N. Engl. J. Med..

[141] (2024). Comparison table: Inhaled drugs for treatment of COPD. Med. Lett. Drugs Ther..

[142] Albert R.K., Schwartz D.A. (2019). Revealing the Secrets of Idiopathic Pulmonary Fibrosis. N. Engl. J. Med..

[143] Huang J., Beyer C., Palumbo-Zerr K., Zhang Y., Ramming A., Distler A., Gelse K., Distler O., Schett G., Wollin L. (2016). Nintedanib inhibits fibroblast activation and ameliorates fibrosis in preclinical models of systemic sclerosis. Ann. Rheum. Dis..

[144] McInnes I.B., Schett G. (2011). The pathogenesis of rheumatoid arthritis. N. Engl. J. Med..

[145] Solomon J.J., Danoff S.K., Woodhead F.A., Hurwitz S., Maurer R., Glaspole I., Dellaripa P.F., Gooptu B., Vassallo R., Cox P.G. (2023). Safety, tolerability, and efficacy of pirfenidone in patients with rheumatoid arthritis-associated interstitial lung disease: A randomised, double-blind, placebo-controlled, phase 2 study. Lancet Respir. Med..

[146] Juge P.A., Lee J.S., Ebstein E., Furukawa H., Dobrinskikh E., Gazal S., Kannengiesser C., Ottaviani S., Oka S., Tohma S. (2018). MUC5B Promoter Variant and Rheumatoid Arthritis with Interstitial Lung Disease. N. Engl. J. Med..

[147] Adegunsoye A., Kropski J.A., Behr J., Blackwell T.S., Corte T.J., Cottin V., Glanville A.R., Glassberg M.K., Griese M., Hunninghake G.M. (2024). Genetics and Genomics of Pulmonary Fibrosis: Charting the Molecular Landscape and Shaping Precision Medicine. Am. J. Respir. Crit. Care Med..

[148] Maher T.M., Schlecker C., Luedtke D., Bossert S., Zoz D.F., Schultz A. (2022). Phase I studies of BI 1015550, a preferential phosphodiesterase 4B inhibitor, in healthy males and patients with idiopathic pulmonary fibrosis. ERJ Open Res..

[149] Pugashetti J.V., Adegunsoye A., Wu Z., Lee C.T., Srikrishnan A., Ghodrati S., Vo V., Renzoni E.A., Wells A.U., Garcia C.K. (2023). Validation of Proposed Criteria for Progressive Pulmonary Fibrosis. Am. J. Respir. Crit. Care Med..

[150] Maher T.M., Stowasser S., Voss F., Bendstrup E., Kreuter M., Martinez F.J., Sime P.J., Stock C. (2023). Decline in forced vital capacity as a surrogate for mortality in patients with pulmonary fibrosis. Respirology.

[151] Buschulte K., Kabitz H.J., Hagmeyer L., Hammerl P., Esselmann A., Wiederhold C., Skowasch D., Stolpe C., Joest M., Veitshans S. (2024). Disease trajectories in interstitial lung diseases-data from the EXCITING-ILD registry. Respir. Res..

[152] Buschulte K., Kabitz H.J., Hagmeyer L., Hammerl P., Esselmann A., Wiederhold C., Skowasch D., Stolpe C., Joest M., Veitshans S. (2024). Hospitalisation patterns in interstitial lung diseases: Data from the EXCITING-ILD registry. Respir. Res..

[153] Blanco I., Tura-Ceide O., Peinado V.I., Barbera J.A. (2020). Updated Perspectives on Pulmonary Hypertension in COPD. Int. J. Chronic Obstr. Pulm. Dis..

[154] Atchley W.T., Kakkera T.K. (2024). Pulmonary hypertension in chronic obstructive pulmonary disease: Current understanding, knowledge gaps and future directions. Curr. Opin. Pulm. Med..

[155] Suresh V., Mih J.D., George S.C. (2007). Measurement of IL-13-induced iNOS-derived gas phase nitric oxide in human bronchial epithelial cells. Am. J. Respir. Cell Mol. Biol..

[156] Hirano T., Matsunaga K., Sugiura H., Minakata Y., Koarai A., Akamatsu K., Ichikawa T., Furukawa K., Ichinose M. (2013). Relationship between alveolar nitric oxide concentration in exhaled air and small airway function in COPD. J. Breath. Res..

[157] Malli F., Gouvani A., Dimeas I., Ladias S., Papathanasiou I.V., Gourgoulianis K.I., Daniil Z. (2021). Exhaled Nitric Oxide (FeNO) in Patients Hospitalized for an Exacerbation of Bronchiectasis and/or COPD: FeNO Levels in COPD and Bronchiectasis. Adv. Exp. Med. Biol..

[158] Maniscalco M., Fuschillo S., Mormile I., Detoraki A., Sarnelli G., Paulis A., Spadaro G., Cantone E. (2023). Exhaled Nitric Oxide as Biomarker of Type 2 Diseases. Cells.

[159] Zeng G., Xu J., Zeng H., Wang C., Chen L., Yu H. (2024). Differential Clinical Significance of FENO(200) and CANO in Asthma, Chronic Obstructive Pulmonary Disease (COPD), and Asthma-COPD Overlap (ACO). J. Asthma Allergy.

[160] Hogman M., Pham-Ngoc H., Nguyen-Duy B., Ellingsen J., Hua-Huy T., Van Nguyen D., Dinh-Xuan A.T. (2024). Measuring exhaled nitric oxide in COPD: From theoretical consideration to practical views. Expert. Rev. Respir. Med..

[161] Christenson S.A., Hanania N.A., Bhatt S.P., Bafadhel M., Rabe K.F., Vogelmeier C.F., Papi A., Singh D., Laws E., Dakin P. (2025). Type 2 inflammation biomarkers and their association with response to dupilumab in COPD (BOREAS): An analysis of a randomised, placebo-controlled, phase 3 trial. Lancet Respir. Med..

[162] Rabe K.F., Celli B.R., Wechsler M.E., Abdulai R.M., Luo X., Boomsma M.M., Staudinger H., Horowitz J.E., Baras A., Ferreira M.A. (2021). Safety and efficacy of itepekimab in patients with moderate-to-severe COPD: A genetic association study and randomised, double-blind, phase 2a trial. Lancet Respir. Med..

[163] Fainberg H.P., Moodley Y., Triguero I., Corte T.J., Sand J.M.B., Leeming D.J., Karsdal M.A., Wells A.U., Renzoni E., Mackintosh J. (2024). Cluster analysis of blood biomarkers to identify molecular patterns in pulmonary fibrosis: Assessment of a multicentre, prospective, observational cohort with independent validation. Lancet Respir. Med..

[164] Brownstein A.J., Mura M., Ruffenach G., Channick R.N., Saggar R., Kim A., Umar S., Eghbali M., Yang X., Hong J. (2024). Dissecting the lung transcriptome of pulmonary fibrosis-associated pulmonary hypertension. Am. J. Physiol. Lung Cell. Mol. Physiol..

[165] Liadaki K., Zafiriou E., Giannoulis T., Alexouda S., Chaidaki K., Gidarokosta P., Roussaki-Schulze A.V., Tsiogkas S.G., Daponte A., Mamuris Z. (2024). PDE4 Gene Family Variants Are Associated with Response to Apremilast Treatment in Psoriasis. Genes.

[166] Rowe S.M., Jones I., Dransfield M.T., Haque N., Gleason S., Hayes K.A., Kulmatycki K., Yates D.P., Danahay H., Gosling M. (2020). Efficacy and Safety of the CFTR Potentiator Icenticaftor (QBW251) in COPD: Results from a Phase 2 Randomized Trial. Int. J. Chronic Obstr. Pulm. Dis..

